# Intramuscular DNA vaccine provides protection in non-human primate and mouse models of SARS-CoV-2

**DOI:** 10.3389/fimmu.2025.1589584

**Published:** 2025-06-06

**Authors:** Subeena Sood, Ebony N. Gary, Majed Matar, Jessica Kim, Casey E. Hojecki, Bryce Warner, Robert Vendramelli, Thang Truong, Alanna Smith, Jennifer Rice, Jeff Sparks, Michael DeSalvo, John Henderson, Joseph A. Rogers, Ankur Sharma, Laurent Pessaint, Carlo A. Iavarone, Darwyn Kobasa, Jean D. Boyer, Stacy Lindborg, Khursheed Anwer

**Affiliations:** ^1^ Imunon, Inc., Lawrenceville, NJ, United States; ^2^ The Vaccine & Immunotherapy Center, The Wistar Institute, Philadelphia, PA, United States; ^3^ National Microbiology Laboratory, Public Health Agency of Canada, Winnipeg, MB, Canada; ^4^ Bioqual, Rockville, MD, United States

**Keywords:** DNA, vaccine, primate, mice, immunology, plasmid, SARS-CoV-2

## Abstract

Nucleic acid vaccine approaches have proven successful in the context of the SARS-CoV-2 pandemic, however challenges with delivery remain. Here we describe PlaCCine, a DNA-based vaccine platform that utilizes a device- and vector-free chemical delivery system. This system includes a DNA plasmid encoding the target antigen and generates robust immune responses, offering significant protection against live viral challenges in both non-human primates and mice. We designed spike plasmid immunogens representing early SARS-CoV-2 strains and found that parental spike PlaCCine vaccination induced SARS-CoV-2 specific cellular and humoral responses in non-human primates and supported significant viral control following challenge. To evaluate immunogenicity and protective efficacy against emerging variants, we further advanced the platform to incorporate the SARS-CoV-2 XBB1.5 variant and observed robust, dose-dependent cellular and humoral responses in mice. When mice were immunized and intranasally challenged with 1×10^5^ TCID_50_ of SARS-CoV-2 XBB1.5 virus, all immunized animals survived the challenge and displayed undetectable lung viral loads. Together these data demonstrate the efficacy of the PlaCCine platform for the delivery of vaccine antigens and support the continued translation of this platform for infectious diseases.

## 1 Introduction

Nucleic acid vaccines have emerged as promising alternatives to conventional vaccine approaches such as protein-based and viral-vectored vaccines. The approval of two mRNA and one DNA vaccine products against COVID-19 has renewed interest in nucleic acid vaccines. While the approval of mRNA vaccines for COVID-19 was timely and beneficial in controlling the pandemic, it also underscores certain limitations of mRNA vaccines, including toxicity (1–3), susceptibility to degradation, short-lived responses, hyperimmune reactions, antigenicity of the lipid carrier, and suboptimal global distribution due to ultra-cold-chain requirements (3). These limitations impact the manufacturing cost and hinder equitable access in developing countries, warranting the development of safer and more effective vaccines. DNA-based vaccines capitalize on the antigen design flexibility of the nucleic acid approach, induce robust cellular responses, support durable antigen expression, and are stable at workable temperatures (4–7). However, challenges with respect to humoral immunogenicity remain with DNA vaccines. Early prophylactic approaches using first-generation DNA delivery were unsuccessful in the clinic primarily due to inadequate antigen production (8). To optimize immunogenicity, adjuvants have been included in some of the vaccine formulations (9).

Several DNA-based prophylactic vaccines have been approved for veterinary use against West Nile virus, infectious hematopoietic necrosis virus, salmon alphavirus type 3, and Avian influenza A virus (10), and one vaccine has been approved for human use against SARS-CoV-2 (11–14). Due to low efficiency of DNA delivery physical delivery devices such as an electroporator or a needle-free jet injector (11–15) have been employed, which despite recent improvements poses challenges in user compliance, additional cost and limited rapid global application. We have recently reported the development of a DNA vaccine that is independent of a device or virus and utilizes a functionalized derivative of a non-ionic triblock copolymer to protect DNA from degradation and facilitate its uptake into muscle by direct injection, which we have termed PlaCCine. It is composed of a central hydrophobic chain attached to two hydrophilic chains with functional molecules. This method leads to plasmid DNA protection from extracellular nucleases, durable gene expression, immune responses, and induces robust protection against SARS-CoV-2 D614G and Delta variants in mice (16). In this study, we demonstrate the immunogenicity and protective activity of these constructs in non-human primates and advance the platform to deliver XBB1.5 variant spike antigens promoting immunogenicity and protection in mice. These data support the continued translation of this approach to larger species in contrast to the decreasing efficiency with increasing body size when using unformulated DNA (17).

## 2 Methods

### 2.1 Vector construction and formulation

The DNA sequences of the SARS-CoV-2 D614G (pvac15), Delta (pvac16), and Omicron XBB1.5 (IMNN-101) variant spike antigens, with a prefusion-stabilizing 2P modification, were codon-optimized and constructed into the plasmid backbone as previously described (16). Small-scale plasmid preparations were routinely produced and characterized for quality attributes before testing. Vaccine plasmids were diluted in PBS to 2 mg/mL and formulated with a functionalized poloxamer and an adjuvant, AlPO_4_, under controlled formulation conditions as previously described (16).

### 2.2 Primate immunogenicity and challenge studies

Primate studies were conducted at BioQual, Inc. (Rockville, MD) in accordance with BioQual IACUC protocol 22-031. Housing and handling of the animals were performed in accordance with the standards of AAALAC International, the Animal Welfare Act as amended, and the Public Health Service Policy, USA. Eighteen adult cynomolgus macaques (3–6 kg) were utilized for these studies. All animals were healthy and free of Filovirus, Herpes B virus, SRV, and TB, and were allocated into three treatment groups and one control group. In the first study, animals were immunized with 1 mg pvac15 expressing the spike antigen of SARS-CoV-2 variant D614G or 100 µg mRNA-1273 on Days 0, 28, and 84. To examine if the PlaCCine approach is effective against a more advanced Delta variant of SARS-CoV-2 at the time, a different set of animals were immunized with pvac16 expressing the spike Delta variant on Days 0, 28, and 56. A 2 mg plasmid dose was also used for pvac16 to monitor gross tolerability associated with higher dosing as determined by physical examination and cage-side observations. Blood was collected before each vaccination, during immunization, and before viral challenge. Body weight and temperature were monitored throughout the study. After the immunization period, animals were challenged intranasally with 1×10^6^ TCID_50_ of the SARS-CoV-2 variant D614G under ketamine sedation. SARS-CoV-2 USA/NY-PV08449/2020 (D614G) stock, NR-53515, was obtained from BEI and expanded at BioQual (Lot# 091620-230) in Vero 76 cells. After virus inoculation, nasal swabs and BAL specimens were collected on Days 1, 2, 4, and 7, followed by euthanasia, necropsy, and tissue collection.

### 2.3 Antibody ELISA for SARS-CoV-2 Spike protein for primate

Nunc MaxiSorp 96-well plates (Thermo Scientific, Cat# 439454) were coated with 50 µL of SARS-CoV-2 RBD protein (Sino Biological Cat# 40592-V08B) diluted to 2 µg/mL in 1X Carbonate-Bicarbonate Buffer (CBB, Sigma, Cat# C3041-50CAP). Plates were incubated overnight at 2-8°C, washed five times with 200 µL with PBS + 0.05% Tween-20 and blocked with 100 µL of PBS + 1% BSA. Test and positive control samples in duplicate were diluted in assay diluent (PBS-Tween20-1% BSA). Plates were incubated for 1 hour at room temperature and washed five times with 200 µL PBS + 0.05% Tween-20. Fifty µL of the secondary detection antibody (Goat anti-Monkey IgG (H+L) secondary antibody, HRP, Invitrogen, PA1-84631) were added at 1:10,000 dilution and plates were incubated for 60 minutes at Room temperature (RT), washed with 200 µL PBS + 0.05% Tween-20 and with 200 µL of PBS. To develop, 100 µL of 1-Step Ultra TMB substrate (SERA CARE, KPL Cat# 5120-0075) were added to each well. The reaction was stopped after 10 min with 50 µL of TMB stop solution (SERA CARE, Cat# 5150-0020). The plates were read within 30 min at 450 nm with a Thermo Labsystems Multiskan spectrophotometer.

### 2.4 Plaque Reduction Neutralization Test in primate

The PRNT assay was conducted in serum samples of test and control subjects before and after vaccination. Vero E6 cells (ATCC, Cat# CRL-1586) were plated in 24-well plates at 175,000 cells/well in DMEM + 10% FBS + Gentamicin. The plates were incubated at 37°C, 5.0% CO_2_ until cells reached 80-100% confluency. Serum samples were heat inactivated and added at 1:10 and 1:3 dilutions. Pseudovirus 30 pfu/well was added to all test and control samples. The plate was incubated at 37°C, 5.0% CO_2_ for 1-hour and 1 mL of 0.5% methylcellulose media was added to each well followed by incubation at 37°C, 5% CO_2_ for three days. The methylcellulose medium was removed, plates were washed once with 1 mL PBS and fixed with 400 μL ice cold methanol per well at -20°C for 30 minutes. After fixation methanol was discarded and monolayers were stained with 250 μL/well of 0.2% crystal violet (20% methanol, 80% dH_2_O) for 30 minutes at room temperature. Plates were then washed once with PBS or dH_2_O and air-dried for 15 minutes. The plaques in each well were recorded and the IC_50_ titers were calculated based on the average plaque density detected in the control wells. A control reference serum (rabbit) with an established titer (5,400 IC_50_) was included in each assay setup to serve as an internal positive control.

### 2.5 Quantitative RT-PCR assay for SARS-CoV-2 RNA

Quantitative RT-PCR assay for viral load was performed on nasal swabs (NS) and bronchoalveolar lavage (BAL) samples in accordance with BioQual standard procedures. The qRT-PCR assay for SARS-CoV-2 subgenomic RNA (sgRNA) utilized primers and a probe specifically designed to amplify and bind to a region of the E gene messenger RNA from SARS-CoV-2, which is not packaged into the virion. The signal was compared to a known standard curve of plasmid and copy number per gram of tissue was calculated. For sample reactions, 45 µL of master mix, 50 µL of reverse transcriptase, and 100 µL of RNase inhibitor (Bioline SensiFAST™ Prob Lo-ROX One-Step Kit), along with a primer pair at 2 µM concentration, were added to 5 µL of sample RNA.

### 2.6 TCID_50_ assay

The TCID50 assay was conducted on BAL and NS samples according to BioQual standard procedures. Vero TMPRSS2 cells (obtained from the Vaccine Research Center-NIAID) were plated at 25,000 cells/well in DMEM + 10% FBS and incubated at 37°C, 5% CO_2_ until 80-100% confluency the next day. Twenty μL of sample were added to the top of the first row in quadruplicate, followed by 10-fold dilutions. Positive control wells (virus stock of known infectious titer in the assay) and negative control wells (medium only) were included in each assay setup. The plates were incubated at 37°C, 5% CO_2_ for 4 days, and the cell monolayers were visually inspected for cytopathic effect (CPE). Non-infected wells had a clear confluent cell layer, while the infected cells showed cell rounding. The presence of CPE was marked on the lab form as a + and the absence of CPE as 0. The TCID_50_ value was calculated using the Reed-Muench formula.

### 2.7 Intracellular cytokine staining and ELISpot assay in primates

Cryopreserved PBMCs were stimulated with matched SARS-CoV-2 spike glycoprotein peptides representing full-length spike antigens (Genscript) for ELISpot or intracellular cytokine staining. Negative controls received an equal concentration of dimethyl sulfoxide without peptides.

### 2.8 Mouse immunogenicity and challenge studies

Mouse studies were performed under Wistar IACUC protocol #201399. Housing and handling of the animals were performed in accordance with the standards of AAALAC International, the Animal Welfare Act as amended, and the Public Health Service Policy, Canada. Animals were housed in the Animal Resource Facility at the Vaccine and Immunotherapy Center, Wistar Institute. Mouse immunogenicity were performed at Wistar Institute and challenge studies at the National Microbiology Laboratory, Winnipeg, Manitoba, CA under protocol H-20-011. Forty female K18-hACE2 mice (6–8 weeks, ~17.5 g) were obtained from Jackson Labs. Mice were immunized with 10 µg, 30 µg, or 50 µg IMNN-101 vaccine on Days 0 and 28, followed by viral challenge on Day 49 with 1×10^5^ TCID_50_ of SARS-CoV-2 Omicron XBB1.5 intranasally. Mice were euthanized four days later, and lung viral loads in tissue were quantified. A second group of mice (n = 5 per group) were monitored for survival to Day 14 after challenge. Antibody ELISA, plaque reduction neutralization test (PRNT) assays and IFN-g ELISpot assays were performed as described previously (10).

### 2.9 Statistical analysis

Statistical analysis was performed using GraphPad Prism 10.1.1 software. All bar graphs, line graphs, scatter plots represent Mean ± SEM. A two-tailed Mann-Whitney test was performed for Primate IgG titers and Neutralization titer between Day 0 and pre-challenge days within respective groups. Samples and animal groups with a p-value ≤ 0.05 were considered statistically significant. For mouse IgG immunology and challenge studies non-parametric Kruskall-Wallis ANOVA, two way ANOVA or Mantel-Cox Log rank analysis was used.

## 3 Results

### 3.1 Induction of humoral and cellular immune responses in PlaCCine-immunized non-human primates

**Figure 1:**
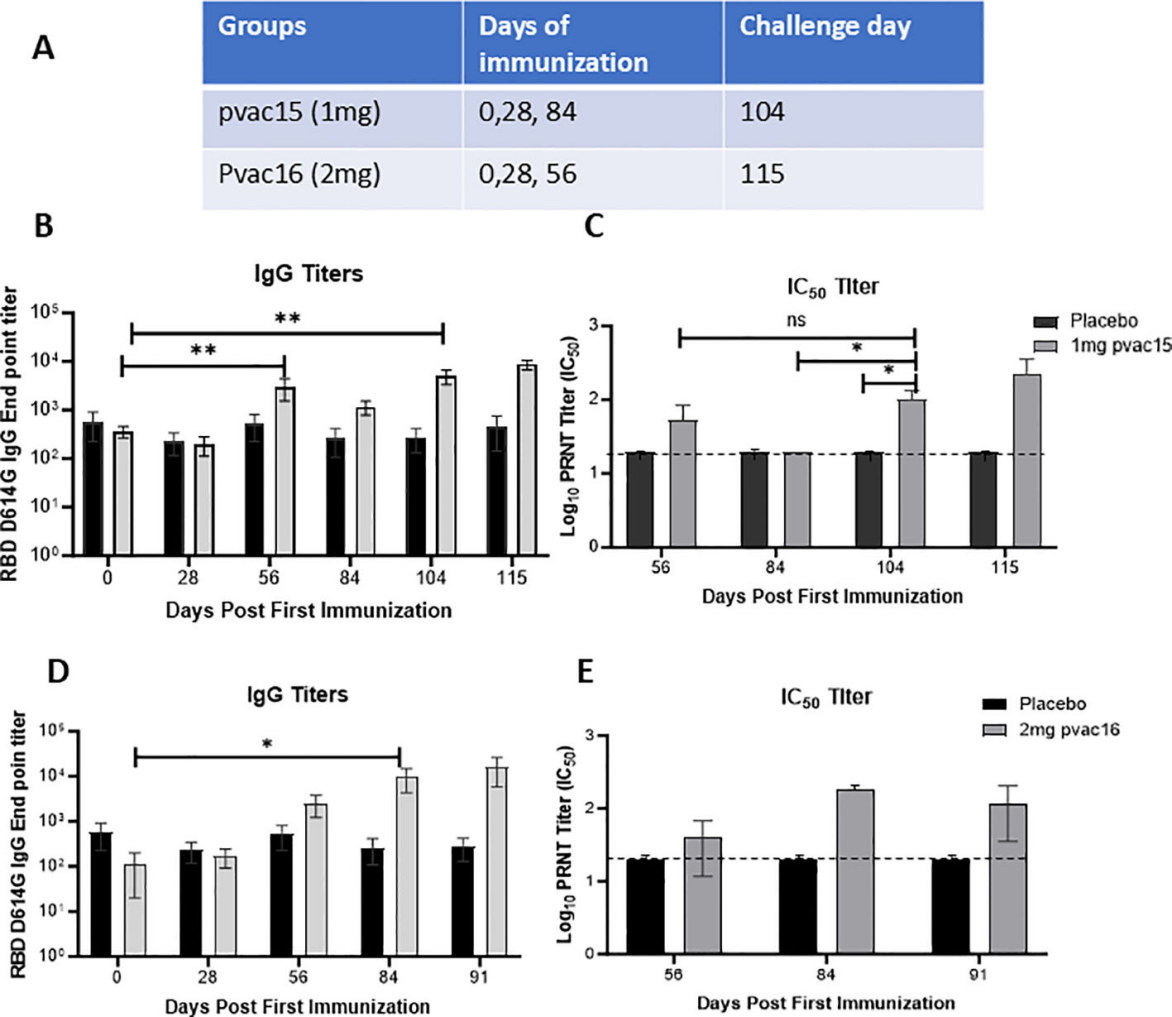
PlaCCine formulation is immunogenic in non-human primates and supports viral control *in vivo*. **(A)** Table showing Cynomolgus macaques were immunized three times with either 1mg pvac15 or 2mg pvac16 in PlaCCine formulation and sera was collected on day 0 pre vaccination, day 28 after one immunization, day 56 after two immunization, day 84 before third immunization, day 104 (pre challenge) and day 115, 7-day post challenge. Longitudinal SARS-CoV-2 spike-binding IgG endpoint titers **(B)**, and live virus neutralizing antibody titers **(C)** among pvac15-immunized macaques. Longitudinal SARS-CoV-2 spike-binding IgG endpoint titers **(D)**, and live virus neutralizing antibody titers **(E)** among pvac16-immunized macaques. Data are representative of 6 animals in pvac15 and 3 animals each in pvac16 and placebo groups. One animal from Day 84 timepoint was excluded from statistical analysis. *p<0.05, **p<0.01 by Mann Whitney nonparametric test. Horizontal bars represent Mean ± SE.

Immunization of cynomolgus macaques (n = 6) with 1 mg pvac15 vaccine targeted against the D614G variant on Days 0, 28, and 84 elicited binding antibody titers against the RBD regions of the SARS-CoV-2 spike antigen. The IgG titer increased from a pre-vaccination mean value of 361 to a peak mean value of 5,020 on Day 104 and a mean value of 8,667 after challenge with live D614G virus (1×10^6^ TCID_50_) administered intranasally (**Figure 1A**). The IgG titers did not decline during the 115-day study period. By comparison, no increase in IgG titers were observed in control animals (n = 6). Pseudovirus neutralizing antibodies were detectable following immunization with the pvac15 vaccine (**Figure 1C**). The mean neutralizing antibody titers during the immunization period for the placebo group were below the detection limit, as shown by the dotted line. The mean IC_50_ titer in pvac15-vaccinated animals was 100 on Days 104 and 228 after viral challenge. Immunization of three macaques with the pvac16 vaccine targeted against the Delta variant on Days 0, 28, and 56 elicited binding antibody titers against the RBD regions of the spike antigen. The IgG titer increased from a pre-vaccination mean value of 110 to a peak mean value of 9,614 on Day 84 and a mean value of 16,008 after challenge with live D614G virus (1×10^6^ TCID_50_) administered intranasally (**Figures 1B**, **D**). As noted with the pvac15 vaccine, the IgG titer in pvac16-immunized animals did not decline during the 91-day study period. Antigen-specific IgG was not observed in control animals (n = 3). Pseudovirus neutralization antibodies were detectable following immunization with the pvac16 vaccine (**Figure 1E**). The mean neutralizing antibody titers for the placebo group were below the limit of detection, as shown by the dotted line. The pvac16-vaccinated animals had a mean IC_50_ titer of 140 on Day 84 and 86.6 post-challenge. The apparent drop in mean neutralizing antibody titers after viral challenge could be the result of a high variability in the group. The variability in end point IgG titers and neutralization titers observed in this study may be attributed to low sample size. Immunization of NHPs with 100 µg mRNA-1273 on Days 0, 28, and 84 elicited binding antibody titers against the spike antigen. The IgG titer increased from a pre-vaccination mean value of 602 to a peak mean value of 170,681 on Day 104, and to a mean value of 136,278 after challenge with live D614G virus (1×10^6^ TCID_50_) administered intranasally. The IgG titer in mRNA-vaccinated animals did not decline during the 115-day study period (data not shown). The mean IC_50_ titer peaked at 6480 on day 104 (pre challenge) with 100ug of mRNA-1273 from 3000 at baseline. The mean IC_50_ titer decreased to 4320 post challenge with D614G virus (1×10^6^ TCID_50_) administered intranasally.

**Figure 2:**
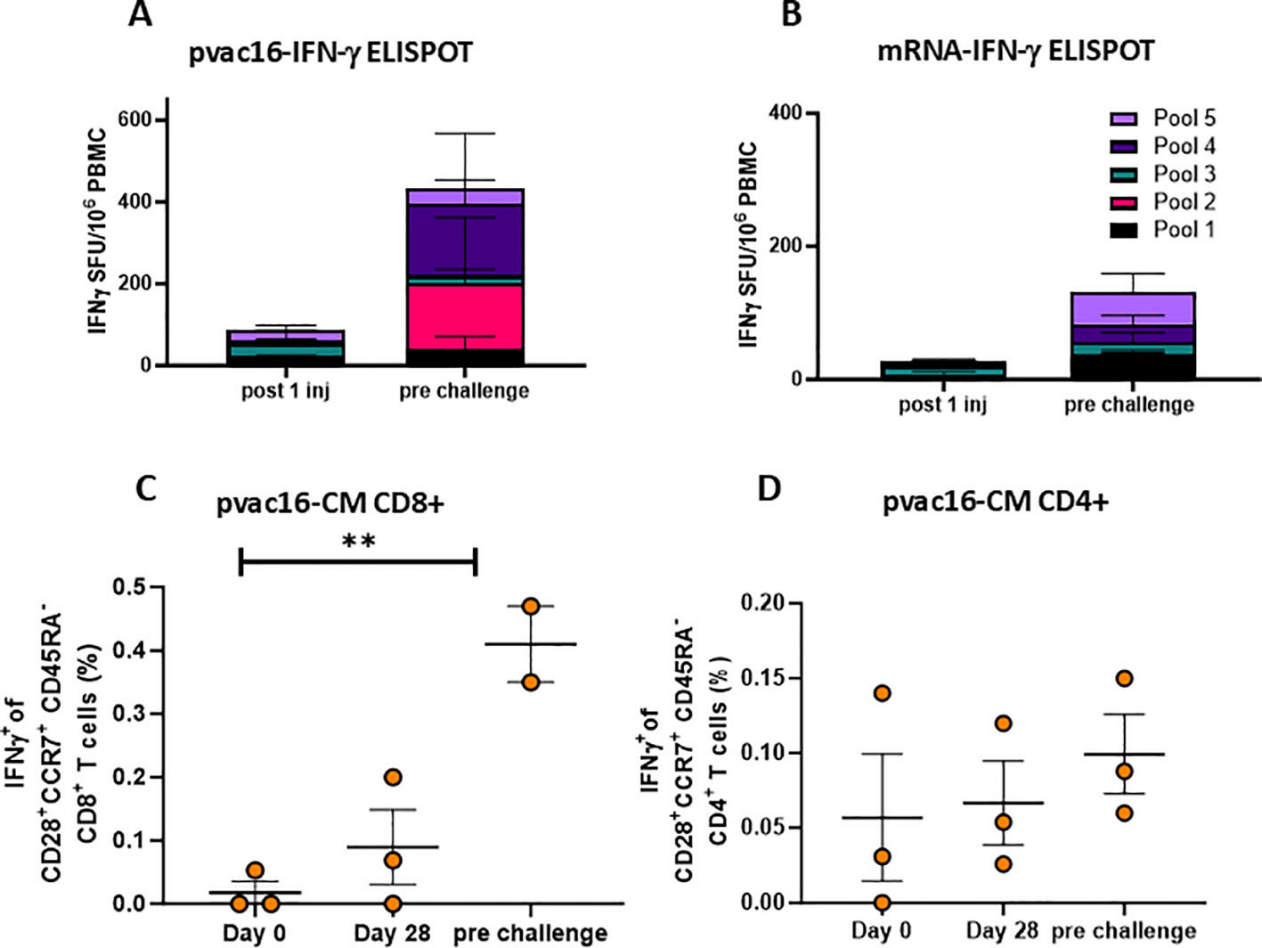
pvac16 induces strong cellular responses in non-human primates. Primates were immunized as in **Figure 1** and interferon-γ secreting cells were enumerated among PBMCs via ELISpot. IFNγ spot forming units (SFU) per million PBMCs following a single immunization (D28) or pre-challenge for **(A)** pvac16, or **(B)** mRNA-immunized NHPs (data not significant). Intracellular cytokine staining (ICS) of spike-specific IFNγ^+^ of CD28^+^CCR7^+^CD45RA^-^ central memory (CM) CD8^+^ **(C)**, and CD4^+^ **(D)** T cells in PBMCs of pvac16 immunized NHPs. ** p<0.01 by Mann Whitney nonparametric test.

PBMCs from pvac16-vaccinated animals were analyzed for antigen-specific IFN-γ-secreting T cells by ELISpot and intracellular cytokine staining (ICS). The pvac16 immunization induced SARS-CoV-2 antigen-reactive T cell responses against all five peptide pools, from 0 SFU/million cells on Day 0 to 174 SFU/million cells before challenge (Day 84; **Figure 2A**), suggesting a durable cellular response. In comparison, mRNA-1273-induced T cell response had a peak value of 48 SFU/million cells with pool 5 before challenge (Day 104; **Figure 2B**). ICS analysis showed an increase in the IFN-γ secreting CD28^+^/CCR7^+^/CD45RA^-^/CD8^+^ central memory T cell population from 0.01% on Day 0 to 0.27% on Day 104 before challenge (**Figures 2C, D**). No significant change was observed in the frequency of IFN-γ secreting CD28^+^/CCR7^+^/CD45RA^-^/CD4^+^ memory T cell population. These data indicate that the pvac16 vaccine elicited a T cell response primarily involving CD28^+^/CCR7^+^/CD45RA^-^/CD8^+^ memory T cells.

### 3.2 Protective efficacy against challenge in non-human primates

**Figure 3:**
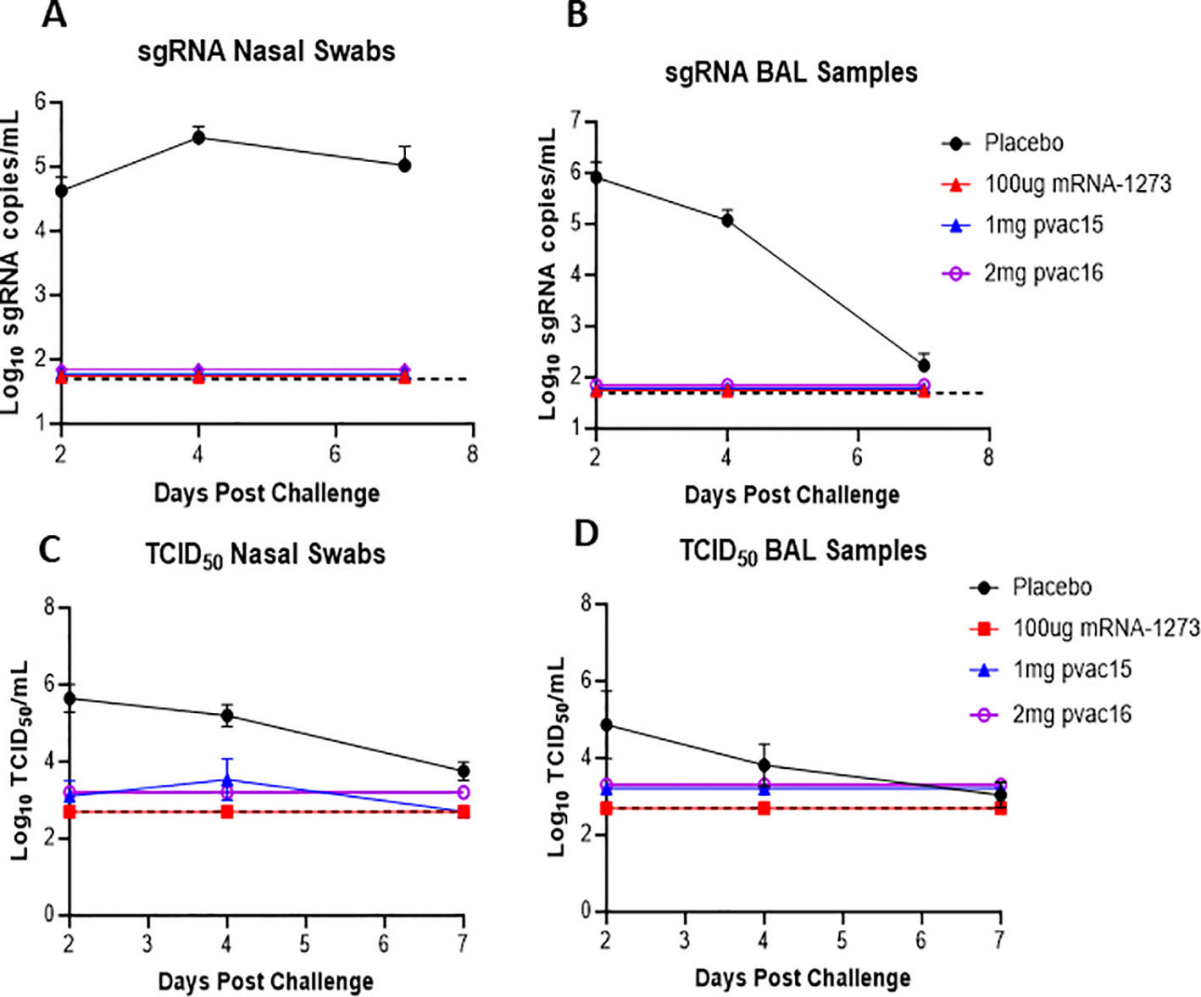
sgRNA in nasal swab and BAL post challenge in non-human primates supports protection post vaccination: Nasal swab sub-genomic RNA over time in placebo, pvac15, pvac16 and mRNA **(A, C)** Bronchoalveolar lavage sub-genomic RNA over time in placebo, pvac15, pvac16 and mRNA **(B, D)**. Data are representative of one experiment with N=6 (pvac15 and mRNA) macaques per group and N=3 (placebo and pvac16) (data not significant). Nasal swab and BAL from SARS-CoV-2 challenged macaques were collected on day 2, 4 and 7 and the amount of sub- genomic RNA copies/ml and TCID_50_/ml was evaluated. The lower detection limit for this assay is 50copies/ml for log RNA copies and 2.7 TCID_50_/mL, represented by the dotted line.

The immunized animals were challenged intranasally with 1x10^6^ TCID_50_ of D614G variant and the viral load was measured in nasal swab (NS) and bronchoalveolar lavage (BAL) on Days 2, 4, and 7 post-challenge using qPCR (sgRNA) and TCID_50_ assays. In control subjects, the viral load in NS remained high throughout the seven-day post-infection period with the highest content (286,433 copies/mL) observed on Day 4 after challenge (**Figure 3A**). In comparison, the nasal viral load in pvac15, pvac16 and mRNA-vaccinated animals was completely reduced to limit of detection within two days post-challenge indicating a rapid clearance. In BAL, the viral load in control macaques was highest on Day 2 (814,720 copies/mL) and progressively declined thereafter approaching near baseline by Day 7. In vaccinated animals, the BAL viral load was completely cleared within two days after challenge (**Figure 3B**). Similar trends in viral clearance were observed in the TCID_50_ assay with > 90% reduction in viral load in pvac15 and pvac16-vaccinated subjects (**Figures 3C, D**). A rapid and complete reduction of viral load in the immunized subjects indicates durable protection with vaccination. The immunization of macaques was observed to be safe as no significant changes were observed in body weight and temperature during the treatment period.

### 3.3 Induction of humoral and cellular immune responses in PlaCCine-immunized BALB/c mice

**Figure 4:**
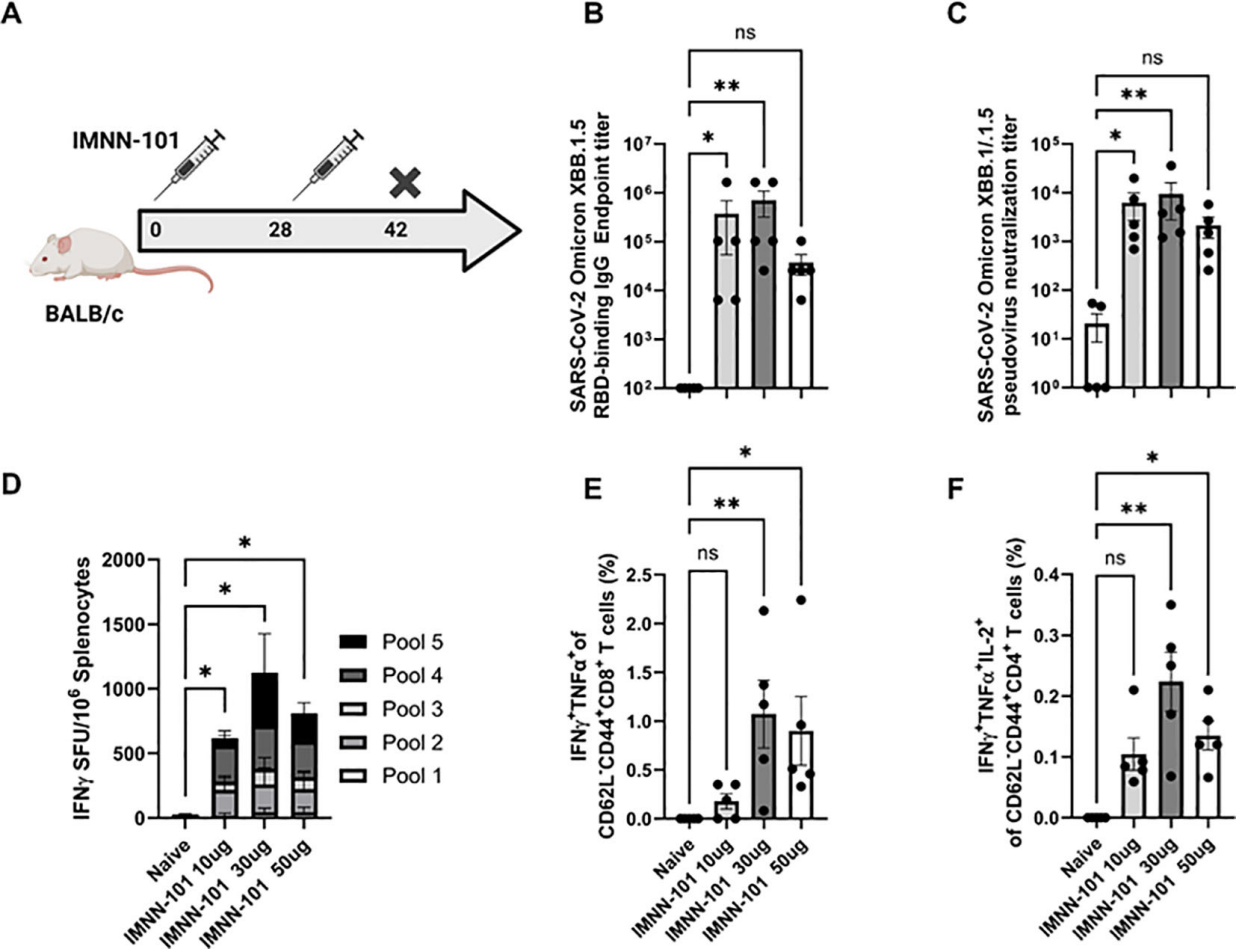
PlaCCine formulated Omicron XBB.1.5 DNA antigen (IMNN-101) support robust humoral and cellular responses in mice/ **(A)** 6-8-week-old BALB/c mice were immunized twice, separated by four weeks with 10, 30, or 50ug of IMNN-101 in PlaCCine formulation and euthanized two-weeks post-final immunization. **(B)** SARS-CoV-2 Omicron XBB.1.5 receptor-binding domain (RBD) binding IgG serum endpoint titers. **(C)** XBB.1.5 spike-pseudotyped virus neutralization titers. **(D)** IFNγ spot-forming units (SFU) in spleens by ELISpot. **(E)** Frequencies of IFNγ^+^ TNFα^+^ CD8^+^ T cells by intracellular cytokine stain (ICS). **(F)** Frequencies of IFNγ^+^TNFα^+^ IL-2^+^ CD4^+^ T cells by ICS. Bars represent group means, symbols represent duplicate **(B, C)** or single **(E, F)** assays for individual animals, and error bars represent SEM **(B–D)** or SD **(E, F)**. *p<0.05, **p<0.01 by or Kruskal-Wallis nonparametric ANOVA **(B, C)**. Data are representative of one experiment with N=5/group. ns (non significant).

As continued variants of concern (VOC) emerged within the Omicron sub-lineage, we applied the PlaCCine technology to deliver SARS-CoV-2 Omicron XBB1.5 specific DNA antigens (IMNN-101). We evaluated IMNN-101 in mouse models before initiating a first-in-human proof-of-concept study. Mice were immunized twice, separated by four weeks with 10 µg, 30 µg, or 50 µg of IMNN-101 and humoral and cellular responses were evaluated two weeks after the second immunization (**Figure 4A**). IMNN-101 immunization increased XBB1.5 RBD-binding IgG and pseudovirus-neutralizing antibodies in serum with similar magnitude across the different groups (**Figures 4B, C**). Cellular responses in immunized animals were observed using IFN-γ ELISpot assay and ICS following stimulation with matched XBB1.5 spike peptides. As shown in **Figure 4D**, the number of IFN-γ spot-forming units in splenocytes was significantly higher in immunized mice compared to naïve controls. Intracellular cytokine staining revealed a significant increase in double cytokine IFN-γ/TNF-α secreting CD8^+^ T cells compared to naïve controls (**Figure 4E**). Similarly, there was an increase in spike-specific IFN-γ/TNF-α/IL-2 secreting CD4^+^ T cells (**Figure 4F**). These data demonstrate that the XBB1.5 PlaCCine candidate, IMNN-101, can induce robust cellular and humoral responses *in vivo*.

### 3.4 Protective effect of IMNN-101 in PlaCCine formulation in transgenic mouse model

**Figure 5:**
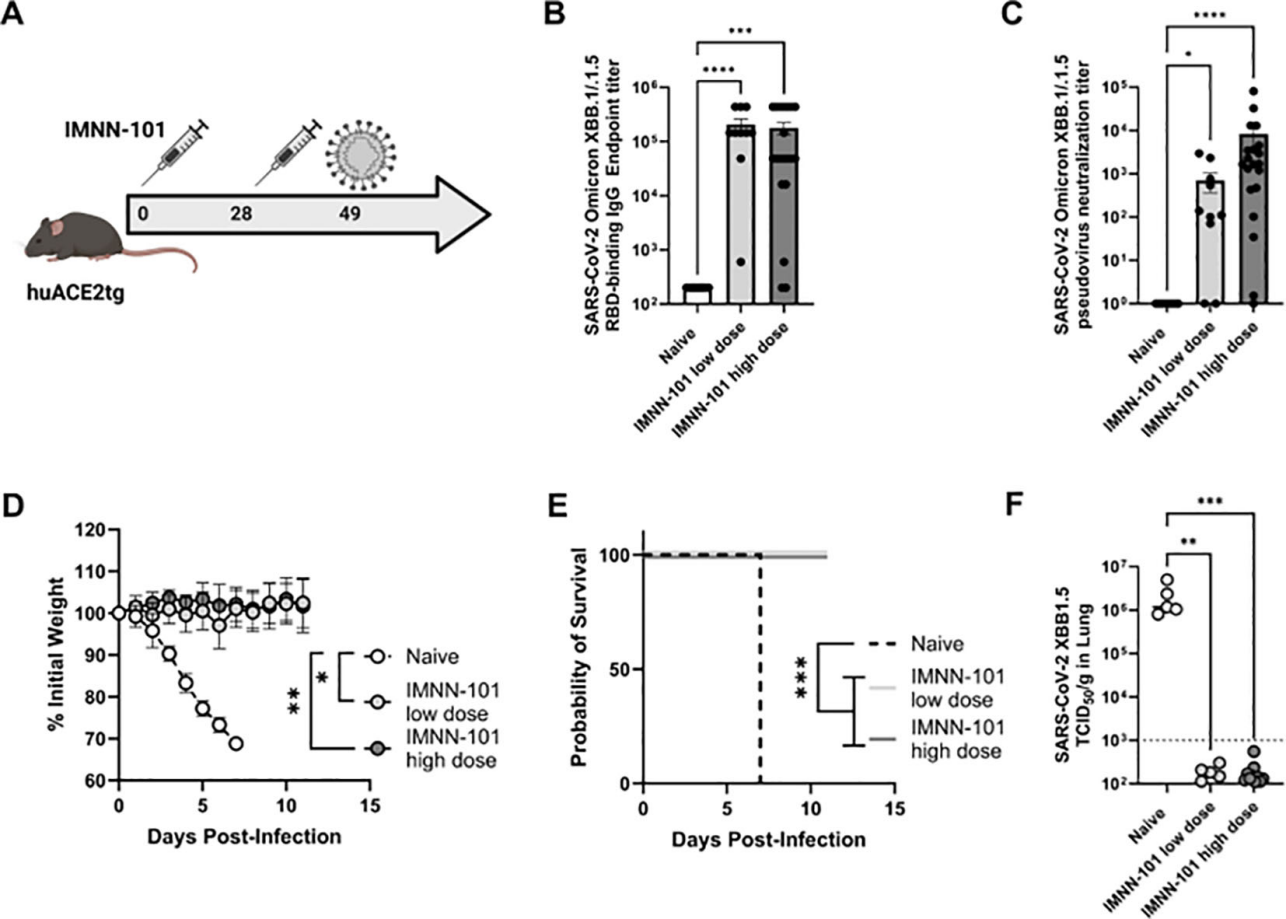
**(A-F)** PlaCCine formulation (IMNN-101) supports complete protection from XBB.1.5 challenge in mice. **(A)** 6–8 week-old human ACE2 transgenic (K18 hACE2Tg) were immunized twice separated by four weeks with plaCCine-formulated synDNA plasmids encoding SARS-CoV-2 Omicron XBB.1.5 spike. 3 weeks post-last vaccine, all animals were intranasally infected with 10^5^ TCID50 SARS-CoV-2 Omicron XBB.1.5. **(B)** XBB.1/XBB.1.5 binding RBD and, **(C)** pseudovirus neutralization capacity of sera at day 21 post-1^st^ immunization. **(D)** Weight loss, and **(E)** survival probability post-challenge. **(F)** XBB.1.5 viral loads at day 4 post-challenge in 5/10 animals per group. *p<0.05, **p<0.01, ***p<0.01, ****p<0.01 Kruskal-Wallis ANOVA, or two way ANOVA or Mantel Cox Log rank analysis **(F)**. Bars represent group means, symbols represent duplicate assays for individual animals, and error bars represent SEM **(B, D)**.

To evaluate protective efficacy IMNN-101 vaccine was administered to human ACE2 transgenic mice as in **Figure 5A** and mice were subsequently challenged intranasally with SARS-CoV-2 XBB1.5 virus. As in BALB/c mice, IMNN-101 supported increased XBB1.5 RBD-binding IgG (**Figure 5B**) and pseudovirus neutralizing (**Figure 5C**) antibodies. All animals were challenged intranasally with 1×10^6^ TCID_50_ SARS-CoV-2 Omicron XBB1.5 and a subset were euthanized four days thereafter to quantify the viral loads in lungs at the peak of infection. All immunized animals were completely protected from morbidity and mortality, while the non-immunized animals lost significant weight (**Figure 5D**) and died within seven days post-challenge (**Figure 5E**). We were unable to detect any replication competent virus in the lungs of immunized animals (**Figure 5F**). These data demonstrate that next-generation PlaCCine-formulated DNA antigens are immunogenic and protective in preclinical challenge models and support the continued advancement of this platform.

## 4 Discussion

PlaCCine-mediated DNA antigen delivery is a unique nucleic acid platform which does not require a device (11) or viral vector (12) to engender cellular and humoral immunity. Compared to mRNA vaccines that require stringent storage and transport requirements, PlaCCines are stable at workable temperatures and are thus suitable for global distribution. Additionally, while a multivalent mRNA vaccine requires each of the contributing mRNAs be manufactured separately, PlaCCine allows for multiple antigen targets in a single plasmid decreasing both production cost and time, particularly critical in managing a pandemic with emerging variants. The proof-of-concept of PlaCCine vaccines for SARS-CoV-2 in mice has been reported previously by our laboratory. In those studies, the immunogenicity and protective efficacy of PlaCCine vaccines against different SARS-CoV-2 variants was demonstrated (16). We now report proof-of-concept of PlaCCine vaccines in non-human primates demonstrating feasibility in larger species, in contrast to the decreasing efficiency with increasing body size when using unformulated DNA (17). Furthermore, we also demonstrated immunogenicity and protective efficacy of our next-generation Omicron XBB1.5 PlaCCine vaccine in preclinical mouse models in support of an ongoing Phase I clinical trial.

The PlaCCine delivery system is a nonionic triblock copolymer of polyoxypropylene and polyoxyethylene covalently modified with a metal chelator to reduce DNA degradation by nucleases. There is evidence of DNA protection from extracellular nucleases by the PlaCCine polymer (16) potentially increasing DNA bioavailability and promoting cellular uptake through cell adsorption and interaction with the lipid cell membrane (18, 19). Poloxamer delivery is associated with increased intracellular membrane permeability and subsequent DNA release from endosomes and transport into nucleus (20, 21). Due to their surfactant property, poloxamers significantly enhance DNA distribution through the tissue and increase DNA bioavailability and interaction with extracellular matrix (18, 20, 22). Poloxamers may also activate selected signaling pathways of selected promoters (CMV, NFkB, p53), resulting in transcription activation of delivered gene in muscles (23).

In this study, vaccination of macaques with PlaCCine vaccines against D614G or Delta variant elicited binding and neutralizing antibodies (nAB). Initially, the animals were immunized with 1 mg pvac15 vaccine (D614G) on Days 0, 28, and 84, and later another group was immunized with 2 mg of pvac16 vaccine (Delta), while placebo served as controls. Both vaccines elicited binding and neutralizing antibodies against their respective pseudoviruses. The nAB responses were similar between the different DNA doses and variant vaccines. Though the humoral responses generated by mRNA vaccination were higher, the magnitude and rates of viral clearance between mRNA and PlaCCine-vaccinated animals were comparable, suggesting that the immune response generated by PlaCCine vaccination is sufficient to provide protection *in vivo*. A similar viral clearance by PlaCCine despite lower antibody response could also be due to cellular responses since antibody responses are not the only potential correlate of vaccines protection. Hence, immune response generated by PlaCCine vaccination provides commensurate protection *in vivo*. This and simple manufacturing and stability at 4 C for up to 12 months (16) suggest PlaCCine as a potential viable vaccine candidate. PlaCCine’s non-lipid composition also offers safety advantages over mRNA vaccines that are often associated with reactogenicity and anaphylaxis (24, 25).

Older device-based approaches can be complex and expensive and may require training and technical expertise thus limiting accessibility and making widespread use challenging (20, 26–28). However, considerable progress has been made in recent years to improve the safety and compliance of devices for gene delivery for human use (29–33). Multiple approaches for delivering SARS-CoV-2 DNA vaccines in non-human primates have been evaluated previously and the use of these approaches has resulted in humoral and cellular responses and protection from viral challenge (34–36). The magnitude and kinetics of immune responses to PlaCCine vaccines in our macaque study are comparable to published reports using electroporation or needle-free injection devices with a similar prime and boost schedule (35, 36). We observed rapid viral clearance after challenge with viral load reaching baseline by two days post-challenge, a faster kinetic than those reported in earlier studies (34, 37). While these differences in challenge outcome are likely due to significant differences in antigen dose, number of immunizations, and challenge viral loads, these data support the continued translation of the PlaCCine platform.

Based on the results from this NHP study and previous mouse studies (16), we developed the IMNN-101 vaccine targeting the XBB1.5 variant, the major VOC at the time these studies were performed, for a proof-of-concept study in humans. This clinical candidate was extensively characterized in normal mice and in ACE2 transgenic mice demonstrating robust humoral and cellular responses and protection against SARS-CoV-2 XBB1.5 virus challenge. The IMNN-101 vaccine is currently being evaluated in healthy volunteers with multiple previous exposures to the SARS-CoV-2 virus or vaccines.

In conclusion, these studies demonstrate immunogenicity and protective efficacy of PlaCCine vaccines against multiple variants of SARS-CoV-2. Thus, PlaCCine vaccines are an attractive potential alternative to other nucleic acid-based vaccine approaches due to their safety, simplicity, low cost of manufacturing, and stability.

## Data Availability

The raw data supporting the conclusions of this article will be made available by the authors, without undue reservation.
